# Metabolic Engineering of the Shikimate Pathway for Production of Aromatics and Derived Compounds—Present and Future Strain Construction Strategies

**DOI:** 10.3389/fbioe.2018.00032

**Published:** 2018-03-26

**Authors:** Nils J. H. Averesch, Jens O. Krömer

**Affiliations:** ^1^Universities Space Research Association at NASA Ames Research Center, Moffett Field, CA, United States; ^2^Department of Solar Materials, Helmholtz Centre for Environmental Research, Leipzig, Germany

**Keywords:** Shikimate pathway, metabolic engineering, aromatics, metabolic modelling, strain construction

## Abstract

The aromatic nature of shikimate pathway intermediates gives rise to a wealth of potential bio-replacements for commonly fossil fuel-derived aromatics, as well as naturally produced secondary metabolites. Through metabolic engineering, the abundance of certain intermediates may be increased, while draining flux from other branches off the pathway. Often targets for genetic engineering lie beyond the shikimate pathway, altering flux deep in central metabolism. This has been extensively used to develop microbial production systems for a variety of compounds valuable in chemical industry, including aromatic and non-aromatic acids like muconic acid, *para*-hydroxybenzoic acid, and *para*-coumaric acid, as well as aminobenzoic acids and aromatic α-amino acids. Further, many natural products and secondary metabolites that are valuable in food- and pharma-industry are formed outgoing from shikimate pathway intermediates. (Re)construction of such routes has been shown by *de novo* production of resveratrol, reticuline, opioids, and vanillin. In this review, strain construction strategies are compared across organisms and put into perspective with requirements by industry for commercial viability. Focus is put on enhancing flux to and through shikimate pathway, and engineering strategies are assessed in order to provide a guideline for future optimizations.

## Introduction

### Importance of Bio-Derived (Aromatic) Building Blocks for the Global Chemical Market

**Figure 1:**
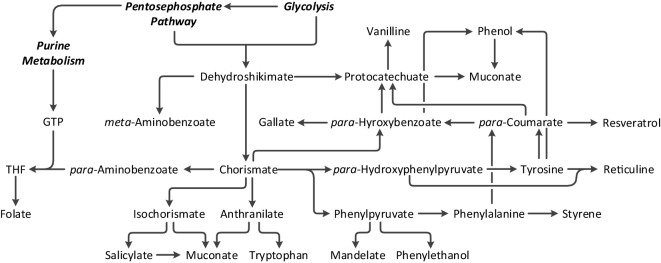
Major branch point intermediates and products associated with and derived from the shikimate pathway by means of metabolic engineering.

The worldwide push to move toward a more sustainable society not only includes the goal to move from fossil fuel dependency toward renewable feedstocks but also aims to maintain and increase standards of living by facilitating the access to pharmaceuticals and securing the availability of foodstuff. Biotechnology is anticipated to be a key to fulfilling these objectives, as biochemical pathways are extremely versatile, giving rise to a wealth of diverse organic compounds (Chen and Nielsen, 2013; Becker and Wittmann, 2015; Becker et al., 2015). It has been predicted that bio-feedstocks could increase to 17% (Webster, 2012) of the global chemical business by 2025, equivalent to 425 billion USD (de Jong et al., 2013; Averesch, 2016), with global demand for biomass derived chemicals exceeding 8.5 Mt by 2023 (Insights, 2016). Unfortunately, to date, the majority of the bio-replacements is hardly cost competitive. This highlights the major hurdle for white biotechnology: outperforming existing chemical synthesis. The cost advantage will strongly depend on yield and titer (ergo the pathway), which comes down to cost of substrates vs. current oil price as well as capital and operational costs, which depend on the rate. Very generalized, the yield of a bioprocess should reach 85% of the theoretical maximum (Peralta-Yahya et al., 2012) and achieve a target product titer higher than 50 g/L (Woodley, 2017) (or close to the solubility limit) at a productivity in the single-figure g/(L × h) range (Woodley, 2017). A minimum specific rate has been defined as 0.01 mol/(g_CDW_ × h) (Averesch et al., 2018). The competition with petrochemistry is less distinct for foodstuffs and especially pharmaceuticals, as in these industries products need to suffice (extremely) high quality standards, and are (often) natural products, which can be scarce and/or are hard to synthesize chemically, which makes them vastly more expensive. Among these, aromatics and aromatics-derived compounds are a very important class with applications in all three areas (chemical, pharmaceutical, and food industries). In biochemistry, aromatic compounds are almost exclusively obtained *via* the shikimate pathway, which not only leads to aromatic amino acids but also gives rise to diverse aromatic precursors allowing the biosynthesis of a great variety of secondary metabolites/natural products (Knaggs, 2003; Kayser and Averesch, 2015; Lai et al., 2017). Research efforts to engineer the shikimate pathway in various ways to establish microbial production systems for bulk and fine chemicals are strong (Thompson et al., 2015; Suástegui and Shao, 2016; Noda and Kondo, 2017), an overview of the compounds that have been bio-derived from the shikimate pathway and are currently bioavailable is given in Figure 1.

### Peculiarities of the Shikimate Pathway in Model Organisms

The shikimate pathway is the central metabolic route leading to formation of tryptophan (TRP), tyrosine (TYR), and phenylalanine (PHE), this pathway exclusively exists in plants and microorganisms (Kayser and Averesch, 2015). It starts with the condensation of intermediates of glycolysis and pentosephosphate-pathway, phosphoenolpyruvate (PEP), and erythrose-4-phosphate (E4P), respectively, which enter the pathway through a series of condensation and redox reactions *via* 3-deoxy-d-arabino-heptulosonate-7-phosphate (DAHP), 3-dehydroquinate (DHQ), 3-dehydroshikimate (DHS) to shikimate. From there the central branch point metabolite chorismate is obtained *via* shikimate-3-phosphate under ATP hydrolysis and introduction of a second PEP (Herrmann, 1995). Recent approaches, especially those reconstructing more complex biosynthetic pathways, rely on *Saccharomyces cerevisiae* as host (Suástegui and Shao, 2016). Therefore, special attention is given to the yeasts’ shikimate pathway. In *S. cerevisiae*, the initial step of the shikimate pathway is catalyzed by two DAHP synthase isozymes (Meuris, 1973; Kunzler et al., 1992), encoded by *ARO4* and *ARO3* and regulated through feedback-inhibition by TYR and PHE, respectively. In *Escherichia coli* three DAHP synthase isozymes exist (*aroF, aroG, aroH*), which are each feedback inhibited by one of the three aromatic amino acids (TYR, PHE, TRP), in contrast the two DAHP synthases of plants are not subject to feedback-inhibition (Herrmann, 1995). In plants and bacteria the subsequent five steps are catalyzed by single enzymes, while in yeast these bioconversions are catalyzed by a pentafunctional protein, a conglomerate of monofunctional domains, which is expressed by the single gene *ARO1* (Duncan et al., 1987). From the central intermediate chorismate the pathway branches off to anthranilate and prephenate leading to aromatic amino acid, para-hydroxybenzoic acid (pHBA) and para-aminobenzoic acid (pABA) synthesis, the latter being a precursor for folate metabolism (Botet et al., 2007).

In *S. cerevisiae*, the gene *ARO7* encodes chorismate mutase which catalyzes the conversion of chorismate to prephenate where the biosynthesis route to TYR and PHE branches off, it is regulated by *GCN4* (Schmidheini et al., 1990). However, this step appears to proceed also spontaneously (Winter et al., 2014; Averesch et al., 2016).

The initial step to TRP biosynthesis is catalyzed by anthranilate synthase, encoded by *TRP2* which forms a multifunctional hetero–oligomeric complex with indole-3-glycerol phosphate synthase encoded by *TRP3*. As well *TRP3* (Prantl et al., 1985) as also *TRP2* (Krömer et al., 2013) may have second functions, as deletions lead to a heavy growth rate reduction regardless of the supplementation of tryptophan.

In *S. cerevisiae*, pABA is a precursor for ubiquinone (coenzyme Q) synthesis (Marbois et al., 2010). Outgoing from chorismate, pABA is synthesized in two subsequent reactions catalyzed by aminodeoxychorismate synthase and aminodeoxychorismate lyase. The aminodeoxychorismate synthase of *S. cerevisiae*, encoded by *ABZ1*, has similarity to *E. coli* pABA synthase components PabA and PabB, indicating that it is a bifunctional enzyme (Edman et al., 1993). In other organisms, like for example *E. coli*, the first step in ubiquinone biosynthesis is formation of pHBA in a single step bioconversion directly from the central shikimate pathway intermediate chorismate. The reaction is catalyzed by chorismate pyruvate lyase, which is encoded by the gene *ubiC* (Nichols and Green, 1992). It is remarkable that pHBA can also be detected in yeast though no chorismate lyase analog has been described to date (Marbois et al., 2010; Pierrel et al., 2010), indicating that either the responsible enzyme has yet to be discovered or the compound is obtained from a different precursor. It has been proposed that this precursor is rather 4-hydroxyphenylpyruvate then catechol, giving rise to a second pathway to ubiquinone synthesis in yeast (Marbois et al., 2010).

Regulation of the shikimate pathway in *S. cerevisiae* is manifold (Lingens et al., 1967) and yet to be fully understood. In general *GCN4*, the transcriptional activator of amino acid biosynthetic genes, controls aromatic amino acid biosynthesis, tightly regulating expression at transcriptional level in response to amino acid starvation (Braus, 1991).

### Industrial Significance of Shikimate Pathway Derived Compounds

Among shikimate pathway derived compounds, feedstocks for polymer industry are most popular, with *cis*,*cis*-muconic acid (ccMA) as biological precursor for the nylon-6,6 building block adipic acid leading the way (Chen and Nielsen, 2013; Polen et al., 2013; Van Duuren and Wittmann, 2014; Xie et al., 2014; Bart and Cavallaro, 2015a,b; Deng et al., 2016). Biotechnological production of ccMA offers a promising alternative to chemical synthesis of adipic acid as conversion to adipic acid is easy and efficient (Polen et al., 2013; Deng et al., 2016; Vardon et al., 2016). Further, ccMA can also be used for the production of terephthalic acid (Burk et al., 2011). Other potential bio-replacements for terephthalic acid are the shikimate pathway intermediates pHBA and pABA (Farlow and Krömer, 2016). pHBA is used mainly in liquid crystal polymers like the high-strength fiber Vectran^®^ and has an estimated market value of approx. 150 million USD per annum (Krömer et al., 2013). It is also the base material for parabens, preservatives in the cosmetic, and pharmaceutical industries (Kluczyk et al., 2002). pABA is a compound with versatile applicability, it is already being used as crosslinking agent for resins and dyes, as a precursor in the pharmaceutical industry and as a therapeutic itself (e.g., for the drug POTABA^®^). It is also a good UV filter as it absorbs UVB radiation (Krömer et al., 2013). Terephthalic acid (Farlow and Krömer, 2016) is a monomer in production of polyethyleneterephthalate. PET is used for packaging as well as clothing and recently also in the auto industry (Research, 2014b). The global market volume for PET packaging was nearing 16 Mt valued at 48.1 billion USD in 2014 (Pira, 2014). This is expected to rise to 19.9 Mt and 60 billion USD in 2019 (Pira, 2014). The global market volume for bio-based PET exceeded 540 kt in 2012 and is expected to rise sharply to more than the 10-fold by 2020 (Research, 2014a). Another potentially pABA-derived polymer precursor is *para*-phenylenediamine, which may potentially be obtained *via*, e.g., Kochi- or Hunsdiecker reaction followed by nucleophilic substitution, as proposed before (Averesch et al., 2016). *para*-Phenylenediamine is (besides terephthalic acid) the second building block of the aramid-fiber Kevlar^®^. Global demand for these materials, which combine high strength with low weight as well as chemical and heat resistance properties, was estimated to 74.5 kt in 2014 and is expected to rise to 110 kt in 2020 with an estimated value of 4.7 billion USD (Markets, 2014).

As a metabolic successor to pABA also production of folates has been pursued in many different bacteria, with lactic acid bacteria leading the way in microbial production of the B vitamin (Sybesma et al., 2003; Patring et al., 2006). As a dietary supplement, this underlines the other great field of application for shikimate pathway derived compounds, which is the food- and pharma-industry. Especially the class of benzylisoquinoline alkaloids (BIAs) are currently in the focus (DeLoache et al., 2015; Galanie et al., 2015), which are derived from tyrosine. BIAs are a class of alkaloids comprised of ca. 2,500 different compounds, with the opioids being the most popular among them. Opioids are indispensable in palliative care and to date still plant-derived, which makes them susceptible to supply difficulties due to environmental factors like climate or disease (Narcross et al., 2016), at the most altering quality and in the worst case resulting in total harvest loss. Further, the demand of approx. 1,000 km^2^ agricultural area for poppy farming to generate the annual demand of ca. 800 t opiates constitutes a potential competition for food crops (Galanie et al., 2015).

Another class of less controversial but nevertheless important plant-derived medical products, which originate from the phenylalanine-branch of the shikimate pathway are the tropan-alkaloids. Scopolamine, the main compound of interest, is an important bulk compound in the semi-synthesis of drugs like Buscopan^®^ and Spiriva^®^. Spiriva was ranked number 13 best-selling drug in 2013 with sales reaching almost 3 billon USD (Brooks, 2014) and continues to be one of the most important medicines. Scopolamine is still exclusively derived from farmed *Duboisia* species (Averesch and Kayser, 2014; Ullrich et al., 2016). Therefore, interest to develop a biotechnological process is high, in order to stabilize supply (Ullrich et al., 2017). Not quite as popular, but still with some pharmaceutical relevance are metabolites of tryptophan. In particular, indole and quinoline alkaloids are used as chemotherapeutic agents (Krivoruchko and Nielsen, 2015).

Another important natural product, which naturally originates from the shikimate pathway from a different branch than the synthetic biology route, is vanillin (Hansen et al., 2009; Kayser and Averesch, 2015). Interestingly, biotechnological production derives vanillin from central shikimate pathway, different to natural production that proceeds *via p*-coumarate (cf. Figure 1). Extraction from vanilla beans can deliver less than 1% of the world’s annual 16 kt demand of vanillin, as it is one of the most popular food aromas with prices up to 1,500 USD/kg for high quality extracts (Evolva, 2014) and a total market value of approx. 650 million USD. It can be made petro-chemically or chemically from lignin waste; however, a fully biological process is favored, due to consumer acceptance (Kaur and Chakraborty, 2013); biotechnological production is run by Evolva.

## Results—Strain Engineering Strategies for Production of Aromatics and Shikimate Pathway Derived Compounds

**Figure 2:**
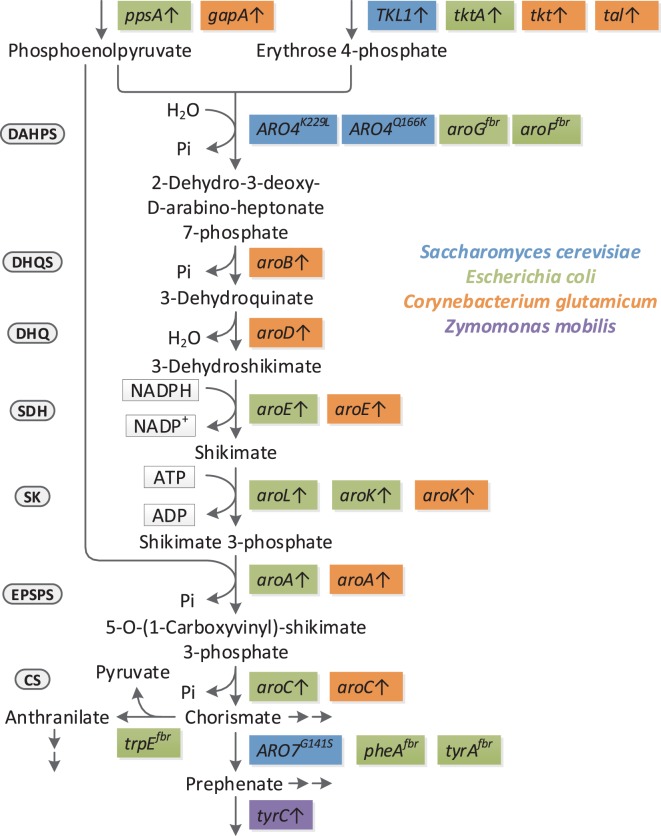
Reported genetic modifications that significantly improve flux to and through the shikimate pathway in the respective context. Only feedback-resistant enzymes and overexpression targets are included, knockouts have not been respected. Highlighted in blue are yeast (in most cases *S. cerevisiae*) genes, green *E. coli* genes, orange *C. glutamicum* genes, and purple *Z. mobilis* genes. With the exception of *aroL* (applied in *S. cerevisiae*), *tyrC* (applied in *E. coli*) and *aroG^fbr^* (applied in *C. glutamicum*) the respective overexpression targets have not been applied outside their native organism. Major enzymes are indicted to the left of the linear part of the pathway; DAHPS, 3-deoxy-d-arabinoheptulosonate 7-phosphate (DAHP) synthase; DHQS, 3-dehydroquinate synthase; DHQ, 3-dehydroquinate dehydratase; SDH, shikimate 5-dehydrogenase; SK, shikimate kinase; EPSPS, 5-enolpyruvylshikimate 3-phosphate synthase; CS, chorismate synthase.

The full spectrum of shikimate pathway derived products is covered; however, only studies outgoing from non-petrochemistry derived carbon-sources are considered, unlike, e.g., production of ccMA in *Pseudomonas putida* from benzoate (Schmidt and Knackmuss, 1984; Bang and Choi, 1995; Choi et al., 1997), as benzoate can hardly be considered a sustainable feedstock. While there are too many studies on shikimate pathway derived products to be compared at once, different reviews have covered this from distinct perspectives, each focusing on certain groups of shikimate pathway derived products (Polen et al., 2013; Rodriguez et al., 2014; Xie et al., 2014; Thompson et al., 2015; Lee and Wendisch, 2017; Narcross et al., 2016; Suástegui and Shao, 2016; Noda and Kondo, 2017; Wang et al., 2018). In contrast to the existing reviews, this review also discusses bio-based production of aromatic compounds progressively by means of certain unique studies, looking at significant modifications that, e.g., increase the precursor availability, channel flux through the shikimate pathway or impact formation of aromatics on a cellular level. For this, a number of values have been calculated, which allow present strain construction strategies to be compared from an industry point of view. In Table 1 the production of different target compounds is compared by used carbon-sources, employed organism and specific strain, genes overexpressed and/or knocked out, final titer, product yield as fraction of theoretical maximum (calculated by metabolic modeling, cf. File S1 in Supplementary Material) productivity. Estimation of specific rates help to further classify the engineering strategies of the respective studies. In addition, Figure 2 gives an overview of all available modifications that enhance carbon flow to and within the shikimate pathway. Finally, a direction toward the next generation of aromatics producing microbial cell factories is given.

**Table 1:** Overview of studies on production of aromatics and aromatics-derived compounds from the shikimate pathway—comparison of target compounds, carbon-sources, organisms and strains, genes overexpressed or knocked out, final titers, and peculiarities of study.

Target compound	Organism/strain	Carbon-source	Pathway-determining intermediate	Gene(s)^b^ (over)expressed	Gene(s) knocked out	Final titer (g/L)	Productivity^c^ (mg/L × h)	Relative carbon-yield^d^ (%)	Characteristics of study	Reference
Shikimate	*Corynebacterium glutamicum*	Glucose	–	*ioT1_Cgl_, glk_Cgl_, ppgk_Cgl_, gapA_Cgl_, tkt_Cgl_, tal_Cgl_, aroG^fbr^_Eco_, aroB_Cgl_, aroD_Cgl_, aroE_Cgl_*	*ptsGHI, hdpA, qsuD, qsuB, aroK*	141	2,937.5	66.18	Fed-batch fermentation, strain can also co-utilize xylose and arabinose	Kogure et al., 2016
Shikimate	*Pichia stipitis*	Glucose	–	*TKT1_Sst_, ARO4^K229L^_Sce_, ARO1^D920A^_Sce_*	–	3.11	25.92	14.96	Shake-flask fermentation, promoter tuning for expression of target genes	Gao et al., 2017
*cis*,*cis*-Muconate	*Escherichia coli*	Glucose	Dehydroshikimate	*tktA_Eco_, aroF^fbr^_Eco_, aroB_Eco_, aroZ_Kpn_, aroY_Kpn_, catA_Aca_*	*aroE*	36.8	766.67	26.13	Fed-batch fermentation	Draths and Frost, 1994; Niu et al., 2002
*cis*,*cis*-Muconate	*E. coli*	Glucose + xylose (2:1)	Dehydroshikimate	ppsAEco, aroGfbrEco, tyrAfbrEco, *hisH^L82R^_Eco_, rpoA^V257F^*,*^L281P^_Eco_*; *shiA_Eco_, aroZ_Kpn_, aroY_Kpn_, catA_Aca_*	*ptsH, ptsI, crr, pheA, tyrR, aroE, ydiB*; *xylA*	4.7	65.28	52.95	Fed-batch bioreactor fermentation, *E. coli* (K12): *E. coli* (BL21) coculture	Zhang et al., 2015
*cis*,*cis*-Muconate	*E. coli*	Glycerol + glucose	Anthranilate	*ppsA_Eco_, tktA_Eco_, aroG^fbr^_Eco_, aroE_Eco_, aroB_Eco_, aroL_Eco_, trpE^fbr^_Eco_, antA_Pae_, antB_Pae_, antC_Pae_, catA1_Ppu_, glnA_Eco_*	*trpD*	0.39	12.19	5.81	Shake-flask fermentation	Sun et al., 2013
*cis*,*cis*-Muconate	*E. coli*	Glycerol + glucose	2,3-Dihydroxybenzoate	*aroL_Eco_, ppsA_Eco_, tktA_Eco_, aroG^fbr^_Eco_, entABC_Eco_, BDC_Kpn_*,^a^*catA1_Ppu_*	*entE*	0.48	10	6.07	Shake-flask fermentation	Sun et al., 2014
*cis*,*cis*-Muconate	*E. coli*	Glucose	2,3-Dihydroxybenzoate	*tktA_Eco_, aroF_Eco_, aroG^fbr^_Eco_, aroB_Eco_, aroL_Eco_, entBA_Eco_, entC_Eco_, entX_Kpn_, catA_Ppu_*	*–*	0.605	8.4	10.99	Shake-flask fermentation	Wang and Zheng, 2015
*cis*,*cis*-Muconate	*E. coli*	Glycerol + glucose	Salicylate	*ppsA_Eco_, tktA_Eco_, aroG^fbr^_Eco_, aroL_Eco_, pchB_Pfl_, entC_Eco_, nahG^opt^_Ppu_, catA1_Ppu_*	*pheA, tyrA*	1.45	30.28	20.82	Shake-flask fermentation, strain based on phenylalanine overproducer NST 74	Lin et al., 2014b
*cis*,*cis*-Muconate	*E. coli*	Glucose	*para*-Hydroxybenzoate	*aroF^fbr^_Eco_, aroE_Eco_, aroL_Eco_, ubiC_Eco_, pobA_Ppu_, aroY_Kpn_, catA_Aba_*	*ptsH, ptsI, crr, pykF*	0.17	2.36	3.09	Shake-flask fermentation	Sengupta et al., 2015
*cis*,*cis*-Muconate	*Pseudomonas putida*	p-Coumarate	*para*-Hydroxybenzoate	*asbF_Bce_, aroY_Ecl_, ecdB_Ecl_, ecdD_Ecl_, catA*	*pcaHG, catRBC*	15.6	213.7	101	Fed-batch bioreactor fermentation	Johnson et al., 2016
*cis*,*cis*-Muconate	*P. putida*	Glucose	Dehydroshikimate	*asbF_Bce_, aroY_Ecl_, ecdB_Ecl_, ecdD_Ecl_, catA*	*pcaHG, catRBC*	4.92	91.11	10.29	Fed-batch bioreactor fermentation	Johnson et al., 2016
*cis*,*cis*-Muconate	*Saccharomyces cerevisiae*	Glucose	Dehydroshikimate	*aroZ_Bth_, asbF_Bth_, aroY_Kpn_, catA_Ara_*	*ARO1*	0.00156	0.0091765	0.0117	Shake-flask fermentation, only partial deletion of *ARO1* (*aroE* analogous domain)	Weber et al., 2012
*cis*,*cis*-Muconate	*S. cerevisiae*	Glucose	Dehydroshikimate	*TKL1_Sce_, ARO4^K229L^_Sce_, aroZ_Pan_*/Pa_5_5120^a^, *aroY_Ecl_*/Ecl_01944^a^, *HQD2_Cal_*	*ZWF1, ARO3, ARO4*	0.14	1.31	1.06	Shake-flask fermentation	Curran et al., 2013
*cis*,*cis*-Muconate	*S. cerevisiae*	Glucose	Dehydroshikimate	*TKL1, ARO1^t^, aroE_Eco_, aroZ_Pan_*/Pa_5_5120,^a^*aroY_Ecl_*/Ecl_01944,^a^*HQD2_Cal_*	*ZWF1, ARO1, ARO3, ARO4*	2.1	8.75	1.93	Fed-batch bioreactor fermentation, shikimate pathway flux improved and redirected through adaptive evolution and truncation of *ARO1*	Leavitt et al., 2017
*cis*,*cis*-Muconate	*S. cerevisiae*	Glucose	Dehydroshikimate	*TKL1_Sce_, ARO4^K229L^_Sce_, ARO1^D(1409^*^,^*^920)A^_Sce_, aroZ_Pan_, aroY_Kpn_, HQD2_Cal_*	*RIC1, ARO1*	0.32	4.45	1.2	Shake-flask fermentation, *in silico* strain design identified novel genetic engineering targets	Suástegui et al., 2017
Catechol	*E. coli*	Glucose	*para*-Hydroxybenzoate	*ubiC_Eco_, pobA_Pae_, aroY_Ecl_*/Ecl_01944^a^	*pheA*	0.63	7.33	8.67	Batch bioreactor fermentation, based on phenylalanine over-producing strain NST 74	Pugh et al., 2014
*para*-Hydroxybenzoate	*Klebsiella pneumonia*	Glucose	Chorismate	*ubiC_Eco_*	–	0.14	10.38	29.34	Shake-flask fermentation, based on triple mutant strain 62-1 (Phe^−^, Trp^−^, Tyr^−^)	Müller et al., 1995
*para*-Hydroxybenzoate	*E. coli*	Glucose	Chorismate	*tktA_Eco_, aroF^fbr^_Eco_, aroB_Eco_, aroL_Eco_, aroA_Eco_, aroC_Eco_, ubiC_Eco_*	–	12	166.67	18.8	Fed-batch bioreactor fermentation	Barker and Frost, 2001
*para*-Hydroxybenzoate	*E. coli*	Glucose + Xylose (2:1)	Dehydroshikimate	aroG^fbr^_Eco_ tyrA^fbr^_Eco_, hisH^L82R^_Eco_, rpo^AV257F, L281P^_Eco_; shiA_Eco_, aroE_Eco_ (aroK_Eco_), aroL_Eco_, aroA_Eco_, aroC_Eco_, ubiC_Eco_	*ptsH, ptsI, crr, pheA, tyrR, aroE, ydiB; xylA*	2.3	23.96	19.92	Fed-batch bioreactor fermentation, *E. coli* (K12): *E. coli* (BL21) coculture	Zhang et al., 2015
*para*-Hydroxybenzoate	*Corynebacterium glutamicum*	Glucose	Chorismate	*xylA_Cgl_, xylB_Cgl_, bglF^317A^_Cgl_, bglA_Cgl_, araBAD_Cgl_, araE_Cgl_, tkt_Cgl_, tal_Cgl_, aroG^S180F^_Eco_, aroCKB_Cgl_, aroD_Cgl_, aroE_Cgl_, aroA_Cgl_, ubiC_Pru_*	*ldhA, qsuB, qsuD, pobA, poxF, pyk, hdpA*	36.6	1,527.38	65.85	Batch bioreactor fermentation, growth arrested culture with multiple integration of key-genes	Kitade et al., 2018
*para*-Hydroxybenzoate	*P. putida*	Glucose	Chorismate	*aroG^fbr^_Eco_, ubiC_Eco_*	*pobA, phA, trpE*	1.73	54.06	23.66	Fed-batch bioreactor fermentation	Yu et al., 2016
*para*-Hydroxybenzoate	*P. putida*	Glucose	Tyrosine	*aroF_Ppu_, PAL_Rto_*	*oprB, pobA, hpd*	0.32	10.59	19.2	Shake-flask fermentation, based on tyrosine over-producing mutant strain S12palB2	Verhoef et al., 2010
*para*-Hydroxybenzoate	*P. putida*	Glycerol	Tyrosine	*aroF_Ppu_, PAL_Rto_*	*oprB, pobA, hpd*	0.23	n.a.^e^	23.51	Shake-flask fermentation, based on tyrosine over-producing mutant strain S12palB2	Meijnen et al., 2011
*para*-Hydroxybenzoate	*P. putida*	Glucose + xylose (1:1)	Tyrosine	*aroF_Ppu_, PAL_Rto_*	*oprB, pobA, hpd*	0.18	0.19	19.85	Chemo-stat bioreactor fermentation, based on mutant strain S12palB2	Meijnen et al., 2011
*para*-Hydroxybenzoate	*Saccharomyces cerevisiae*	Glucose	Chorismate	*ubiC_Eco_*	*ARO7*	0.09	0.82	1.12	Fed-batch bioreactor fermentation	Krömer et al., 2013
*para*-Hydroxybenzoate	*S. cerevisiae*	Glucose	Chorismate	*TKL1_Sce_, ARO4^K229L^_Sce_, ubiC_Eco_*	*ARO7, ZWF1, CDC19, PYK2*	0.15	2.08	0.92	Shake-flask fermentation, dynamic control of overexpression/knock-down targets	Williams et al., 2015
*para*-Hydroxybenzoate	*S. cerevisiae*	Glucose	Chorismate	*ARO4^K229L^_Sce_ aroL_Eco_, ubiC_Eco_*	*ARO7, TRP3*	2.9	29	4.06	Fed-batch bioreactor fermentation	Averesch et al., 2017
Gallate	*E. coli*	Glucose + glycerol + yeast extract	para-hydroxybenzoic acid	ppsA_Eco_, tktA_Eco_, aroG^fbr^_Eco_, aroL_Eco_, ubiC_Eco_, pobA^Y385F,T294A^_Pae_	–	1.27	35.17	n.d.^y^	Shake-flask fermentation based on strain BW25113	Chen et al., 2017
*para*-Aminobenzoate	*E. coli*	Glucose	Chorismate	*aroF^fbr^_Eco_, pabAB_Cef_, pabC_Eco_*	*–*	4.8	100	32.31	Fed-batch shake-flask fermentation	Koma et al., 2014
*para*-Aminobenzoate	*Corynebacterium glutamicum*	Glucose	Chorismate	*aroG^fbr^_Eco_, aroB_Cgl_, aroD_Cgl_, aroE_Cgl_, pabAB_Cca_, pabC_Xbo_*	*ldhA*	43	897.12	28.73	Shake-flask fermentation	Kubota et al., 2016
*para*-Aminobenzoate	*S. cerevisiae*	Glucose	Chorismate	*ABZ1_SceAWRI1631_*	*ARO7, TRP3*	0.03	0.19	0.45	Fed-batch bioreactor fermentation	Krömer et al., 2013
*para*-Aminobenzoate	*S. cerevisiae*	Glycerol + ethanol	Chorismate	ARO4K229L*_Sce_, ABZ1_SceAWRI1631_, ABZ2_SceQA23_*	*ARO7, TRP3*	0.22	2.09	3.02	Fed-batch bioreactor fermentation	Averesch et al., 2016
*meta-A*minobenzoate	*E. coli*	Glucose	Dehydroshikimate	*aroG^fbr^_Eco_, tyrA^fbr^_Eco_, hisH^L82R^_Eco_, rpoA^V257F^*^,^*^L281P^_Eco_*; *shiA_Eco_, pctV*	*pheA, tyrR, aroE, ydiB*	0.048	0.333	0.404	Shake-flask fermentation, *E. coli* (K12): *E. coli* (XL10-Gold) coculture	Zhang and Stephanopoulos, 2016
*ortho*-Aminobenzoate	*P. putida*	Glucose	Chorismate	*aroG^fbr^_Eco_, trpE^fbr^_Eco_G*	*trpDC*	1.54	22.65	7.66	Fed-batch bioreactor fermentation	Kuepper et al., 2015
Tryptophan	*E. coli*	Glucose	*ortho*-Aminobenzoate	*tktA_Eco_, ppsA_Eco_, aroG^fbr^_Eco_, trpE^fbr^_Eco_*	*trpR, tnaC*	40.2	1,005	32.82	Fed-batch bioreactor fermentation	Shen et al., 2012
R-Mandelate	*E. coli*	Glucose	Prephenate/phenylpyruvate	*aroF^fbr^_Eco_, pheA^fbr^_Eco_, hmaS_Aor_, hmo_Sco_, dmd_Rgr_*	*tyrA, tyrB, trpE, aspC*	0.68	28.33	16.32	Shake-flask fermentation	Sun et al., 2011
Phenylethanol	*Kluyveromyces marxianus*	Glucose	Prephenate/phenylpyruvate	*aroG^fbr^_Kpn_, ARO10_Sce_, ADH2_Sce_*	–	1.3	18.06	16.92	Shake-flask fermentation, based on evolved strain resistant to p-fluorophenylalanine	Kim et al., 2014
Phenylethanol	*S. cerevisiae*	Glucose	Prephenate/phenylpyruvate	*ARO4^K229L^_Sce_, ARO7^G141S^_Sce_*	*ARO3, TYR1, ARO8*	0.408	n.d.^y^	5.31	Shake-flask fermentation	Romagnoli et al., 2015
Phenylalanine	*E. coli*	Glucose	Prephenate/phenylpyruvate	*aroK_Eco_, aroL_Eco_, aroA_Eco_, aroC_Eco_, pheA_Eco_, tyrB_Eco_*	–	62.47	1,301.46	48.12	Fed-batch bioreactor fermentation, enzyme concentrations balanced to optimize production	Ding et al., 2016
*para*-Coumarate	*S. cerevisiae*	Glucose	Tyrosine	*aroL_Eco_, ARO4^K229L^_Sce_, ARO7^G141S^_Sce_, TAL_Fjo_*	*PDC5, ARO10*	1.93	26.81	7.91	Fed-batch deep-well plate-fermentation	Rodriguez et al., 2015
Resveratrol	*S. cerevisiae*	Glucose + ethanol	*para*-Coumarate	*Acc1p^S659A^*^,^*^S1157A^_Sce_, ARO4^K229L^_Sce_, ARO7^G141S^_Sce_, TAL_Hau_, 4CL1_Ath_, VST1_Vvi_*	–	0.53	5.21	0.25	Fed-batch bioreactor fermentation	Li et al., 2015
Styrene	*E. coli*	Glucose	*trans*-Cinnamate	*PAL2_Ath_, FDC1_Sce_*	–	0.26	8.97	5.6	Shake-flask fermentation, based on phenylalanine over-producing strain NST 74	McKenna and Nielsen, 2011
Styrene	*S. cerevisiae*	Glucose	*trans*-Cinnamate	*ARO4^K229L^_Sce_, PAL2_Ath_, FDC1_Sce_*	*ARO10*	0.029	0.604	0.464	Shake-flask, strain based on phenylalanine over-producing mutant	McKenna et al., 2014
Tyrosine	*E. coli*	Glucose	Prephenate/*para*-hydroxy-phenylpyruvate	*tyrA_Eco_*	*pheLA*	0.18	4.29	15.67	Shake-flask fermentation, based on phenylalanine over-producing strain NST 37	Olson et al., 2007
Tyrosine	*E. coli*	Glucose	Prephenate/*para*-hydroxy-phenylpyruvate	*ppsA_Eco_, tktA, aroG^fbr^_Eco_, tyrA^fbr^_Eco_*	*tyrR*	9.7	440.91	17.76	Fed-batch bioreactor fermentation	Lütke-Eversloh and Stephanopoulos, 2007
Tyrosine	*E. coli*	Glucose	Prephenate/*para*-hydroxy-phenylpyruvate	*pheA_Eco_, tyrC_Zmo_*	–	3	111.11	11.49	Bioreactor fermentation	Chávez-Béjar et al., 2008
Tyrosine	*E. coli*	Glucose	Prephenate/*para*-hydroxy-phenylpyruvate	*aroG^fbr^_Eco_, tyrA^fbr^_Eco_, rpoA^V257F^*^,^*^L281P^_Eco_*	*tyrR, pheA*	13.8	383.33	20.9	Fed-batch bioreactor fermentation, based on high-performance strain from engineering & high-throughput screening (*rpoA*14^R^)	Santos et al., 2012
Reticuline	*E. coli*	Glycerol	Tyrosine/*para*-hydroxy-phenylpyruvate	*ppsA_Eco_, tktA_Eco_, aroG^fbr^_Eco_, tyrA^fbr^_Eco_*, Mlut_10320^a^, Pp_2552^a^, Rcs0337^a^, *NCS1_Cja_*, 6OMT*_Cja_*^a^, *CNMT_Cja_*, 4OMT*_Cja_*^a^	*tyrR*	0.046	0.45	0.316	Shake-flask fermentation	Nakagawa et al., 2011
Reticuline	*S. cerevisiae*	Glucose	Tyrosine/*para*-hydroxy-phenylpyruvate	*ARO4^K229L^_Sce_, CYP76AD1^W13L^^F309L^_Bvu_*, Pp_2552^a^, NCS*_Pso_*^a^, 6OMT*_Pso_*^a^, CNMT*_Pso_*^a^, *CYP80B1_Eca_*, 4OMT*_Pso_*^a^	–	0.0000806	0.00083958	0.00055	Shake-flask fermentation, developed and use of enzyme-coupled biosensor for improvement of tyrosine hydroxylase	DeLoache et al., 2015
Reticuline	*S. cerevisiae*	Glucose	Tyrosine/*para*-hydroxy-phenylpyruvate	*TKL1_Sce_, ARO4^Q166K^_Sce_, ARO7^T227L^_Sce_, ARO9_Sce_, ARO10_Sce_*, Pp_2552,^a^*NCS1_Cja_*, 6OMT*_Pso_*,^a^ CNMT*_Pso_*,^a^*CYP80B1_Eca_*,^a^ 4OMT*_Pso_*^a^PTPS*_Rno_*,^a^*sepR_Rno_, tyrH^WR^_Rno_*, PCD*_Rno_*,^a^ QDHPR*_Rno_*^a^	*ZWF1*	0.000192	0.002	0.0025	Deep-well plate-fermentation, heterologous metabolic route for BH4 regeneration allows use of mammalian tyrosine hydroxylase, ascorbate stimulates activity	Trenchard et al., 2015

*Though comprehensive this table is not complete, merely milestones and most recent representative studies were selected*.

*^a^unconventionally-, systematically- or un-named gene (locus tag)*.

*^b^Indices indicate species of origin (organism code): Aba, *Acinetobacter baumannii*; Aca, *Acinetobacter calcoaceticus*; Aor, *Amycolatopsis orientalis*; Ara, *Acinetobacter radioresistens*; Ath, *Arabidopsis thaliana*; Bce, *Bacillus cereus*; Bth, *Bacillus thuringiensis*; Bvu, *Beta vulgaris*; Cal, *Candida albicans*; Cca, *Corynebacterium callunae*; Cef, *Corynebacterium efficiens*; Cgl, *Corynebacterium glutamicum*; Cja, *Camellia japonica*; Eca, *Eschscholzia californica*; Ecl, *Enterobacter cloacae*; Eco, *Escherichia coli*; Fjo, *Flavobacterium johnsoniaeu*; Hau, *Herpetosiphon aurantiacus*; Kpn, *Klebsiella pneumonia*; Pae, *Pseudomonas aeruginosa*; Pan, *Podospora anserine*; Pfl, *Pseudomonas fluorescens*; Ppu, *Pseudomonas putida*; Pru, *Providencia rustigianii*; Pso, *Papaver somniferum*; Rgr, *Rhodotorula graminis*; Rno, *Rattus norvegicus*; Rto, *Rhodosporidium toruloides*; Sce, *Saccharomyces cerevisiae*; Sco, *Streptomyces coelicolor*; Sst, *Scheffersomyces stipitis*; Vvi, *Vitis vinifera*; Xbo, *Xenorhabdus bovienii*; Zmo, Zymomonas mobilis*.

*^c^Determined from the quotient of the final titer and the production time (unless values were given in the respective publications)*.

*^d^Achieved carbon-yield as fraction of theoretical maximum carbon-yield, calculation explained in detail in File S1 in Supplementary Material*.

*^e^“not available”—value could not be determined due to insufficient coverage of experimental results-data in the respective study*.

*^y^“not determined”—value could not be determined with satisfactory confidence due to lack of sustainable assumptions in the specific case*.

*In Nakagawa et al. (2011) and Sun et al. (2011) product-concentrations, which were referred to as yields, where handled as titers*.

### Production of ccMA

*De novo* production of ccMA from glucose *via* the shikimate pathway has been pioneered in *E. coli* (Draths and Frost, 1994). The pathway was established in two steps outgoing from dehydroshikimate (DHS), *via* protocatechuate (PCA) and catechol by means of the *aroZ* gene from *Klebsiella pneumoniae* encoding DHS dehydratase, the *aroY* gene from *K. pneumoniae* encoding PCA decarboxylase and the *catA* gene from *Acinetobacter calcoaceticus* encoding catechol 1,2-dioxygenase. The level of the entry metabolites to shikimate pathway was increased by overexpressing *tktA* and carbon flux was increased by means of a feedback-inhibition resistant DAHP synthase *aroF^fbr^* alongside overexpression of the *E. coli* native *aroB*. Further, blocking of the pathway below DHS by deletion of the DHS dehydrogenase gene *aroE*, directed carbon flux to ccMA. The optimized process showed a high maximum titer of 36.8 g/L and its productivity, which is close to reaching g/(L × h), remains so far unrivaled (Niu et al., 2002). The yield, however, stays far behind the theoretical maximum (cf. Table 1).

Production of ccMA *via* a different branch of the shikimate pathway in *E. coli* utilizing glucose and glycerol could only achieve 390 mg/L ccMA (Sun et al., 2013). There, ccMA was derived downstream of chorismate from anthranilate on the TRP synthesis branch of the shikimate pathway. This was done by cloning an anthranilate 1,2-dioxygenase from *Pseudomonas aeruginosa* (encoded by the genes *antA antB antC*, cloned as an operon) and catechol 1,2-dioxygenase from *Pseudomonas putida* (*catA1*) in *E. coli*. TRP biosynthesis was blocked and overexpression of glutamine synthase resulted in a strengthened glutamine regeneration system favoring anthranilate formation in combination with a feedback-inhibition resistant anthranilate synthase (*trpE*) and overexpression of PEP synthetase (*ppsA), tktA, aroG^fbr^, aroE, aroB, aroL*, and *trpE^fbr^*. Two more variations of this pathway for production of ccMA have been developed by this group, both involve the isomerization of chorismate to isochorismate. The first one proceeds *via* 2,3-dihydroxybenzoate (Sun et al., 2014), the other one *via* salicylate (Lin et al., 2014b). The routes delivered respective titers of 480 and 1,500 mg/L. In the first case this was achieved using a strain with a deletion in *entE*, and further engineering to channel flux to chorismate, as described in another study (Lin et al., 2013). Overexpression of the *E. coli* genes *entABC* (coding for 2,3-dihydro-2,3-DHBA dehydrogenase, isochorismatase, and isochorismate synthase) along with a *K. pneumoniae* 2,3-DHBA decarboxylase and the same catechol 1,2-dioxygenase as in the previous study (Sun et al., 2013) completed the pathway. In the second case an “off the shelf” phenylalanine over-producing *E. coli* strain NST 74 (ATCC31884) with deletions in *pheA* and *tyrA* was amended with the same modifications to increase precursor abundance as described previously (Lin et al., 2013). Overexpression of the genes for isochorismate synthase from *E. coli* (*entC*), isochorismate pyruvate lyase from *Pseudomonas fluorescens* (*pchB*), an optimized salicylate 1-monoxygenase from *Pseudomonas putida* (*nahG^opt^*) and the catechol 1,2-dioxygenase, as described before (Sun et al., 2013), lead to ccMA production. The pathway *via* salicylate was the only one to reach a meaningful yield around 1/5th of the theoretical maximum, which is a performance close to the record by the Frost-Lab. Also productivity was highest *via* salicylate, however, orders of magnitude lower than the respective benchmark (Frost-Lab).

A “hybrid” of the DHS-pathway and the routes *via* chorismate to ccMA leads from chorismate *via* pHBA to PCA, from here the steps coincide with the pathway *via* DHS. It was shown that in *E. coli* PCA and catechol can be produced this way, with the highest yield being that of catechol at a titer of 451 mg/L (Pugh et al., 2014). Here, strain construction was also based on the phenylalanine over-producing strain NST 74. Overexpression of chorismate lyase, encoded by *ubiC*, initiated the pathway, a pHBA hydroxylase (*pobA*) from *P. aeruginosa* and a protocatechuate decarboxylase from *Enterobacter cloacae* completed it. To further increase flux to the target compounds the chorismate mutase/prephenate dehydratase (*pheA*) was disrupted. Recently the complete pathway from glucose to ccMA was established (Sengupta et al., 2015). The engineered *E. coli* strain was capable of producing almost 170 mg/L ccMA. The strain had deletions in the genes *ptsH, ptsI, crr*, and *pykF* to boost PEP levels available to the shikimate pathway. Further the *E. coli* DAHP synthase, shikimate 5-dehydrogenase, shikimate kinase II and chorismate lyase (*aroF^fbr^, aroE, aroL*, and *ubiC*) were overexpressed along with a *pobA* from *P. putida, aroY* from *K. pneumoniae*, and *catA* from *Acinetobacter* sp. ADP1 coding for 4-hydroxybenzoate hydrolyase, protocatechuate decarboxylase, and catechol 1,2-dioxygenase, respectively. Despite the extensive genetic engineering, the yield as well as the specific rate of product formation were comparatively low (0.5 mg/g_CDW_ × h), hinting at limitations in one or more steps of this pathway.

In a different approach to sustainable ccMA production, carbon-flux enters the shikimate pathway starting from lignin-derived species. In the respective study, a ccMA titer of 13.5 g/L was obtained (Deng et al., 2016). This was done by engineering the metabolism of a *P. putida* KT2440 strain: in a pathway that uses downstream aromatic amino acid metabolism intermediates like *p*-coumarate as substrates, a route to ccMA was constructed that partially coincides in the last steps with the route *via* pHBA. The same approach was followed in another study where in addition PCA decarboxylase activity was enhanced by expressing associated proteins to produce ccMA from lignin and glucose in *P. putida* KT2440, leading to titers of 15.6 and 4.9 g/L (Johnson et al., 2016). This way complete conversion of *p*-coumarate into ccMA was achieved at the highest specific rate.

Initial *de novo* production of ccMA in *S. cerevisiae* was established by following a similar strain construction strategy as in the first approach in *E. coli* (Draths and Frost, 1994): partial deletion of *ARO1* (the *aroE* analogous domain) blocked the conversion of 3-dehydroshikimate into shikimate, while a DHS dehydratase from *Bacillus thuringiensis* (*aroZ*), a PCA decarboxylase from *K. pneumoniae* (*aroY*), and a catechol 1,2-dioxygenases from *Acinetobacter radioresistens* (*catA*) composed the pathway (Weber et al., 2012). The bottleneck here appeared to be the first step: PCA levels no higher than 7 mg/L could be detected, which limited the final titer to only 1.56 mg/L (Weber et al., 2012). In an analogous approach the DHS dehydratase was taken from *Podospora anserina* and the PCA decarboxylase from *Enterobacter cloacae* (Curran et al., 2013). A catechol 1,2-dioxygenase from *Candida albicans* completed the pathway. In addition, knockout of *ARO3* and over expression of the feedback-resistant *ARO4^K229L^* increased overall flux to aromatics. Further optimizations were deletion of *ZWF1* and overexpression of *TKL1*. The former intended to increase flux to the shikimate pathway in order to increase conversion of G6P to E4P, by blocking the channeling of G6P into the oxidative branch of the pentose phosphate pathway. The latter favored the conversion of pentoses into E4P by increased transketolase levels. In this study, a final ccMA titer of 141 mg/L could be achieved. In a follow up study the previous strain was improved using a biosensor for aromatic amino acids (Leavitt et al., 2016), performing adaptive laboratory evolution to improve flux through the shikimate pathway. Flux was then redirected, following a similar approach as in Weber et al. (2012): by deleting the *ARO1* and expressing a truncated version thereof, flux was redirected from dehydroshikimate toward ccMA, while auxotrophy was prevented by attenuated expression of the *E. coli aroE*. With the final strain a titer of 2.1 g/L could be reached in a fed-batch bioreactor experiment (Leavitt et al., 2017), while also the yield was improved it, however, remained far (10-fold) behind the studies on *E. coli*, reaching only about a fiftieth of the theoretical maximum. Compared to *E. coli*, also productivities and specific rates are orders of magnitude lower (cf. Table 1).

### Production of pHBA

Initially bio-based production of pHBA was established in plants, like *N. tabacum* (Siebert et al., 1996; Köhle et al., 2003) or sugarcane (McQualter et al., 2005). Microbial production of pHBA from glucose was first reported using *K. pneumonia*; deriving it from chorismate a final titer of 120 mg/L was reached (Müller et al., 1995). Due to the low amount of carbon-source this nevertheless corresponds to a significant yield (cf. Table 1). This was done by introducing the *E. coli* chorismate lyase, encoded by *ubiC*, on a plasmid into a *K. pneumonia* strain deficient in the ability to produce aromatic amino acids. While the specific rate could only be roughly estimated (cf. File S1 in Supplementary Material), it nevertheless ranks in an order of magnitude among the highest (double-digit mg/g_CDW_ × h range), indicating the organism’s potential as producer for aromatics. As a foremost reason its pathogenicity has probably stopped its exploitation for this purpose and the research community has focused on safer microbial hosts. Bacterial production of pHBA has been enabled in *Pseudomonas putida*, reaching a titer of 0.32 (Verhoef et al., 2010) and 1.73 g/L (Yu et al., 2016), while both groups reached comparable yields approaching 1/4th of the theoretical maximum, the latter reached a fivefold higher productivity. The superiority of the more recent approach may be attributable to the shorter pathway, deriving pHBA directly from chorismate, rather than downstream of tyrosine. Fermentative production of pHBA in *E. coli* has been patented (Johnson et al., 1998) and reported (Barker and Frost, 2001) deriving the compound *via* chorismate from glucose, where a final concentration of 12 g/L was achieved at similar yields (approx. 1/5th of the theoretical maximum) and rates as in *Pseudomonas*. This was done by overexpression of the feedback-inhibition resistant DAHP synthase *aroF^fbr^* as well as elevation of expression levels of other enzymes in the pathway (*tktA, aroB, aroL, aroA*, and *aroC*). The conversion of chorismate to pHBA was accomplished by the overexpression of *ubiC*. It was found that the DAHP synthase was feedback inhibited by pHBA while DAHP synthase overexpression reduced chorismate lyase expression levels (Barker and Frost, 2001). An active transport of the aromatic out of the cell (Van Dyk et al., 2004) may contribute to achieving these comparatively high titers of extracellular pHBA. More recently, the same *E. coli* coculture system that was used for production of ccMA from glucose and xylose, was also used for production of pHBA (Zhang et al., 2015). The dehydroshikimate over-producing strain was complemented with a strain where the downstream shikimate pathway was engineered for conversion of dehydroshikimate into pHBA *via* chorismate. Due to the diluting nature of the fed-batch process only a titer of 2.3 g/L was reached, the yield, however, was comparable to the previous approaches. The highest production of pHBA was achieved in *C. glutamicum* in a growth-arrested culture with multiple integration of key-genes (Kitade et al., 2018). A titer of 36.66 g/L was reached, at almost 2/3rd of the theoretical maximum yield (which represents a new record and is on one level together with the shikimate-producing strain of the same group), with the second highest rate of aromatics production (1.5 g/h) to date. This was achieved through extensive metabolic engineering, in both, the shikimate pathway itself and throughout the central metabolism. In particular, the final production strain had deletions in the dihydroxyacetone phosphatase and pyruvate kinase (*hdpA* and *pyk*) to re-route carbon flux, as well as multiple integrations of the chorismate synthase, shikimate kinase, 3-dehydroquinate synthase and a feedback-inhibition resistant DAHP synthase (*aroCKB* and *aroG^fbr^*). Further, besides other modifications (deletion of *ldhA, qsuB, qsuD, pobA, poxF*, and overexpression of *xylA, xylB, bglF^317A^, bglA, araBAD, araE, tkt, tal, aroD, aroE, aroA*), it featured a new feedback-inhibition resistant *ubiC* from *Providencia rustigianii*.

Production of pHBA in *S. cerevisiae* was first demonstrated by overexpressing the *ubiC* gene from *E. coli* in a strain where drainage of flux away from chorismate was prevented by deletions of the *ARO7* and *TRP3* genes, abolishing the biosynthesis of aromatic amino acids. The titer of the a proof-of-principle study reached 90 mg/L (Krömer et al., 2013). Production of pHBA in *S. cerevisiae* was revived using a substantially different approach, where gene silencing was regulated by a dynamic circuit (Williams et al., 2015). This allowed application of *in silico* determined knockouts (Averesch and Krömer, 2014), which are lethal when applied constitutively. The circuit autonomously triggers gene expression at a high population density, and was linked with an RNA interference module to enable target gene silencing. This was used to control flux through the shikimate pathway for the production of pHBA. Dynamic RNA repression allowed gene knock-downs, which were identified by elementary flux mode analysis as highly productive but with low biomass formation, to be implemented after a population growth phase. In particular, silencing of *ARO7, ZWF1* and the two pyruvate kinase genes *CDC19* and *PYK2* along with overexpression of *TKL1, ARO4^K229L^* and *ubiC* resulted in a pHBA titer of 0.15 g/L. In a further study an *S. cerevisiae* strain previously engineered to channel flux to chorismate (Averesch et al., 2016) was optimized for pHBA formation. There, constitutive deletions of *ARO7* and *TRP3*, as well as expression of *ARO4^K229L^* and *aroL* were used to increase flux through the shikimate pathway. Further, *ubiC* was screened against the mutated version *ubiC^CCSS^*, which supposedly has enhanced solubility (Holden et al., 2002). Surprisingly *ubiC* still performed better (Averesch et al., 2017), also kinetic limitations appeared to be not as profound as reported (Holden et al., 2002). The strain was then used to stepwise develop a production process; in a fed-batch bioreactor a final titer of 2.9 g/L could be reached, at a productivity of almost 30 mg/(L × h) and a yield that reached 4% of the theoretical maximum.

In addition to being an intermediate for production of ccMA, pHBA can also be an intermediate for production of gallic acid. This was recently shown by engineering the *p*-hydroxybenzoate hydroxylases (*pobA*) from *Pseudomonas aeruginosa* (containing the mutations Y385F and T294A) in a way that enabled formation of gallic acid from pHBA. With additional overexpression of *ppsA, tktA, aroG^fbr^, aroL*, and *ubiC* the *E. coli* strain reached a product titer of 1.27 g/L (Chen et al., 2017). In another study, a pathway to phenol was established *via* pHBA, which is an alternative to the route *via* tyrosine and shortens the pathway (Thompson, 2017).

In this context a synthetic variation of the shikimate pathway for production of terephthalic acid is also worth mentioning—it utilizes the same biochemical conversion steps as for production of pHBA *via* chorismate, with the only difference from the natural shikimate pathway being that it originates from a different precursor: Outgoing from a compound analogous to erythrose 4-phosphate, where the –OH group at the C2 position is replaced by –CH_3_, namely “2-hydroxy-3-methy1-4-oxobutoxy phosphonate,” this pathway leads to the di-acid (terephthalic acid) rather than the hydroxyl-acid (pHBA) (Osterhout et al., 2013).

### Production of Aminobenzoates

Production of pABA in *S. cerevisiae* was first demonstrated by overexpressing an *ABZ1* gene coding for aminodeoxychorismate synthase from the wine yeast strain AWRI1631 in an *ARO7* and *TRP3* knockout strain, resulting in a final titer of 34 mg/L (Krömer et al., 2013). After the initial study in *S. cerevisiae*, recently also enhanced production of pABA in *E. coli* has been reported, reaching maximum titers of 4.8 g/L (Koma et al., 2014). This was accomplished by employing the *E. coli* feedback-inhibition resistant DAHP synthase *aroF^fbr^* and aminodeoxychorismate lyase *pabC* genes in combination with a *Corynebacterium efficiens* aminodeoxychorismate synthase *pabAB*, which combines both domains of the bifunctional pABA synthase in one protein. pABA production in *S. cerevisiae* was revamped by re-engineering a strain that incorporated the feedback-resistant *ARO4^K229L^* as well as deletions in the *ARO7* and *TRP3* genes, in order to channel flux to chorismate. This strain was used to screen different *ABZ1* and *ABZ2* genes for pABA production. In glucose-based shake-flask fermentations the highest titer was reached when overexpressing the *ABZ1* and *ABZ2* genes from the wine yeast strains AWRI1631 and QA23, respectively. *In silico* metabolic modeling indicated a metabolic advantage for pABA production on glycerol and combined glycerol–ethanol carbon-sources. This was confirmed experimentally and in a fed-batch bioreactor experiments pABA a titer of 237 mg/L was reached (Averesch et al., 2016). In parallel pABA production in *C. glutamicum* was accomplished; screening different genes for pABA formation in a similar way as in the *S. cerevisiae* study, *pabAB* from *Corynebacterium callunae, pabC* from *Xenorhabdus bovienii* were identified as most efficient (Kubota et al., 2016). In a strain incorporating an *aroG^fbr^* from *E. coli* and a deletion in *ldhA* (lactate dehydrogenase), while overexpressing the native *aroB, aroD*, and *aroE* genes (3-dehydroquinate synthase, 3-dehydroquinate dehydratase, shikimate 5-dehydrogenase) a pABA titer of 43 g/L was reached. The yield reached almost 30% of the theoretical maximum and also the rate approached the threshold for industrial feasibility, underlining the meaningfulness of this host-engineering targets combination.

Microbial production of the pABA isomer anthranilate (*ortho*-aminobenzoic acid, oABA) has also been established, in particular by engineering *Pseudomonas putida*: A feedback-resistant DAHP synthase (*aroG^fbr^*) was overexpressed in combination with a feedback-resistant anthranilate synthase (*trpE^fbr^*) while the anthranilate phosphoribosyl transferase (*trpD*), indole-3-glycerol phosphate synthase (*trpC*) and chorismate mutase (*pheA*) were knocked out in order to redirect carbon-flux. The highest oABA titer of 1.54 g/L was, however, obtained in a strain that still carried the chorismate mutase (Kuepper et al., 2015).

Recently also the third isomer, meta-aminobenzoic acid (mABA), has become bioavailable and a biological production system has been demonstrated (Zhang and Stephanopoulos, 2016). This was accomplished based on the previously developed *E. coli*–*E. coli* coculture system for production of ccMA and pHBA by the Stephanopoulos Lab (see above). One strain accumulates DHS while the second one converts it to the aromatic compound of interest (in this case mABA), accomplished by a PLP-dependent 3-aminobenzoate synthase (*pctV*). Comparing the obtained titer, yield, and most importantly rate of mABA production to the previous studies on production of ccMA and pHBA, which are, respectively, 198- and 73-fold higher, it becomes clear that PctV must be the bottleneck.

### Production of Aromatic Amino Acids and Phenylpyruvate-Branch Derived Products

Although only a minor biotechnological product, the same basic engineering strategy was used for production of TRP in *E. coli* (Shen et al., 2012). By overexpressing *tktA* (transketolase) and *ppsA* (PEP synthase) in combination with *aroG^fbr^* and *trpE^fbr^* 40.2 g/L could be produced. The corresponding yield and productivity are approaching the defined requirements for an industrial process (approx. 1/3rd of guide values), however, the mediocre specific rate indicates shortcomings in the strain construction.

Production of mandelic acid in *E. coli* was established by introducing a heterologous pathway consisting of the genes *hmaS, hmo*, and *dmd*, encoding for hydroxymandelate synthase (*Amycolatopsis orientalis*), hydroxymandelate oxydase (*Streptomyces coelicolor*) and mandelate dehydrogenase (*Rhodotorula graminis*). In addition, flux through the shikimate pathway was redirected by deletions of *tyrA* (chorismate mutase/prephenate dehydrogenase), *tyrB* (aromatic amino acid aminotransferase), *trpE* (anthranilate synthase) and *aspC* (aspartate aminotransferase) as well as introduction of *aroF^fbr^* and *pheA^fbr^*. The resulting final titers were 0.74 g/L S-mandelic acid and 0.68 g/L R-mandelic acid, respectively (Sun et al., 2011).

Production of phenylethanol, which is mainly used as a fragrance, but has recently also been identified as a next generation biofuel, has so far mostly been established via the Ehrlich-pathway, which degrades PHE. Since PHE itself is a metabolic end product and a compound with commercial valuable, this approach can hardly be considered industrially relevant (Zhang et al., 2014b). *De novo* production of phenylethanol has been established in different organisms, including *E. coli* (Kang et al., 2014) and different yeasts (Kim et al., 2014; Romagnoli et al., 2015). Strain construction strategies mostly focus on enhancing phenylpyruvate decarboxylase and alcohol dehydrogenase activities, while inhibiting PHE formation by deletion of the respective transaminase (*ARO8*), thus blocking the entry of the Ehrlich-pathway. In combination with the usual feedback-resistant DHAP synthase (and chorismate mutase) respective titers in the range of 1.3 and 0.37 g/L could be reached (Kim et al., 2014; Romagnoli et al., 2015). It is noteworthy that in a study on production of pABA in *S. cerevisiae* [(Averesch et al., 2016), see above], in glucose batch cultures phenylethanol titers of almost 0.1 g/L were obtained, which exceeded the target product, even though the chorismate mutase (*ARO7*) was knocked out (Averesch, 2016). This was attributed to a flux overflow that degraded chorismate into prephenate and phenylpyruvate (Winter et al., 2014). In respect of this, it might be worthwhile for future engineering strategies to focus more on increasing overall flow to and through shikimate pathway rather than phenylethanol formation itself. In particular, in a study on production of *p*-coumaric acid in *S. cerevisiae* in addition to the same feedback-resistant DAHP synthase *ARO4^K229L^* and chorismate mutase *ARO7^G141S^* the shikimate kinase was identified as major bottleneck and overexpression of *aroL* in the *S. cerevisiae* strain significantly improved production, reaching a final titer of 1.93 g/L (Rodriguez et al., 2015). The same pathway has also been extended toward production of resveratrol, where a titer of 0.53 g/L could be obtained in a fed-batch setup from glucose and ethanol (Li et al., 2015). More recently, coumaric acid producing yeast strains were also utilized for production of different flavonoids (Rodriguez et al., 2017b). In a study on transcriptional changes in *p*-coumaric acid over-producing *S. cerevisiae* strains, downregulation of amino acid and sugar transporters was observed. Knockouts of some of these (transporters of aromatic amino acid, *TAT1*, and polyamines, *TPO1*) resulted in an over 40% increased production (Rodriguez et al., 2017a).

Extensive work has been done on the production of PHE, as it is a valuable amino acid with diverse applications in food- and pharma-industry. In the most notable recent work the optimum concentrations of six enzymes (*aroK, aroL, aroA, aroC, pheA*, and *tyrB*) along the shikimate pathway were adjusted in *E. coli* allowing an impressive final titer of 62.47 g/L to be reached (Ding et al., 2016), with a yield of roughly half of theoretical maximum and the third highest productivity of an aromatic product. This is even more impressive when considering how far downstream of the main pathway the compound is derived and underlines the importance for identification of bottlenecks in order to balance the pathway and tailor specific strain construction strategies.

PHE is a precursor for *trans*-cinnamic acid, which leads to the production of aroma compounds like cinnamaldehyde as well as cinnamyl- and hydrocinnamyl-alcohol, demonstrated in *S. cerevisiae* (Gottardi et al., 2017). Further, styrene can be produced *via trans*-cinnamate; in *E. coli* this was established based on the PHE over-producing strain NST 74, in which expression of *PAL2* (phenylalanine ammonia lyase) from *Arabidopsis thaliana* and *FDC1* (*trans*-cinnamic acid decarboxylase) from *S. cerevisiae* lead to styrene production of 0.26 g/L (McKenna and Nielsen, 2011). The same group also showed production in *S. cerevisiae*, where first a PHE over-producing mutant was developed through metabolic evolution, in which *ARO1, ARO2, ARO3*, and *ARO8* appeared to be significantly upregulated. Then the same *PAL2* and *FDC1* genes were overexpressed, together with *ARO4^K229L^*, resulting in a styrene titer of 29 mg/L (McKenna et al., 2014).

### Production of Tyrosine and Derived Products

Biotechnological production of benzylisoquinoline alkaloids (BIAs) and precursor thereof has become accessible with the first study reporting heterologous *de novo* reticuline biosynthesis in *E. coli* (Nakagawa et al., 2011). In the respective study, the *tyrR* gene was disrupted, and feedback-inhibition-resistant versions of the DAHP synthase (*aroG^fbr^*) and chorismate mutase (*tyrA^fbr^*) were introduced, while the *ppsA* and transketolase (*tktA*) were exogenously introduced to enhance tyrosine production prior to the BIA synthetic pathway.

Biosynthesis of reticuline has also been reported in *S. cerevisiae* by two independent laboratories, following different approaches (DeLoache et al., 2015; Trenchard et al., 2015) to establish the BIA synthetic pathway. Both studies apply a feedback-inhibition resistant DAHP synthase, while the latter also includes the *ZWF1* knockout, overexpression of the transketolase (*TKL1*), aromatic aminotransferase II and phenylpyruvate decarboxylase (*ARO9* and *ARO10*) and a feedback-inhibition resistant chorismate mutase (interestingly the uncommon *ARO7^T227L^* is used—also the DAHP synthase, *ARO4^Q166K^*, is a different one than used by most other researchers, cf. Box 1). The full pathway to opioids consist of more than a handful of steps downstream of chorismate, which are likely to be rate limiting at this stage; however, in future product formation may benefit from increased availability of the pathway’s substrates. Especially channeling flux to tyrosine may be important in respect of the previously discussed flux overflow in *S. cerevisiae* (Winter et al., 2014; Averesch et al., 2016). For example, for production of TYR in *E. coli*, it was reported that product formation was enhanced when expressing *tyrC* from *Zymomonas mobilis*, a feedback-inhibition-insensitive cyclohexadienyl dehydrogenase in combination with *pheA*, the chorismate mutase-prephenate dehydrogenase native to *E. coli*, rather than using *tyrA* (Chávez-Béjar et al., 2008). Transfer of these genes into a yeast-based production system for compounds derived from TYR (e.g., BIAs) may be worthwhile, potentially in combination with the deletion of *PHA2*, this may channel flux more efficiently to TYR.

Box 1 **Feedback-inhibition resistant DAHP synthases.**

Another point to consider when optimizing and balancing flux entering the shikimate pathway is the existence of various mutated DAHP synthases for both, *E. coli* (Kikuchi et al., 1997; Koma et al., 2012; Zhang et al., 2014a) and *S. cerevisiae* (Hartmann et al., 2003; Helmstaedt et al., 2005; Luttik et al., 2008), many of which feature altered feedback-regulation. In *E. coli*, AroG contributes by far the most (approx. 80%) to total DAHP synthase activity and is inhibited by PHE, while AroF (approx. 20% activity) and AroH are inhibited by TYR and TRP, respectively (Lin et al., 2014a). In *S. cerevisiae* ARO3p and ARO4p are tightly regulated by TYR and PHE, respectively, while the latter is also feedback-inhibited by high concentrations of PHE and TRP (Kunzler et al., 1992). For *E. coli* a variety of genes exist that encode feedback-inhibition resistant versions of all three isozymes (the most popular ones being *aroF^P148L^, aroG^P150L^, aroG^D146N^, aroF^D147N^*, and *aroG^F209S^*), while for *S. cerevisiae* so far only feedback-inhibition resistant versions of ARO4p have been described (*ARO4^K229L^* and the rarely applied *ARO4^Q166K^*). All of this needs to be taken into consideration when developing strain construction strategies toward, e.g., aromatic amino acid derived compounds or/and when including deletions that require the supplementation of certain aromatic amino acids. Therefore, a holistic comparison of the performance and the effects of the different feedback-inhibition resistant DAHP synthases in different organisms would be especially important. It could especially be interesting to see how *E. coli* genes perform in *S. cerevisiae* and potentially also vice-versa. This has partially been covered when comparing *ARO4^K229L^* vs. *aroG^L175D^, aroG^S180F^*, and *aroF_NST74_*, for production of *p*-coumarate in *S. cerevisiae* (Rodriguez et al., 2015), where *ARO4^K229L^* appeared to be most effective.

## Conclusion

### Final Thoughts on Available Targets for Genetic Engineering

Considering that in general the reactions of the shikimate pathway are thermodynamically favored (Averesch, 2016), it can be concluded that limitations are mostly kinetic and/or regulatory. This is supported by a study showing the performance of the shikimiate pathway in *S. cerevisiae* to be greatly dependent on the type strain (Suástegui et al., 2016). In reverse this means that the shikimate pathway is most likely tightly regulated, hence the greatest challenge will be to overcome this, rather than the fine tuning of individual reactions at the final step to the target product. While many approaches have been made to increase production of certain individual products from shikimate pathway, all applying different strain engineering strategies (cf. Figure 2), only a few target optimizations beyond the reactions of the pathway. In addition to what has been described in Box 2, two commonly applied targets beyond the shikimate pathway are overexpression of the transketolase and deletion of the glucose-6-phosphate dehydrogenase. The latter, once a popular knockout target in yeast (*ZWF1*) is rarely applied anymore, a trend that is in accordance with our findings from *in silico* metabolic modeling (Averesch, 2016): we found that the knockout rarely benefits the yield (opposing to its original intention); especially in case of reducing pathways it negatively impacts redox cofactor availability (NADPH production from PPP). This is supported by our previous study on production of pHBA (described above) (Williams et al., 2015), where knock-down of *ZWF1* had an adverse effect on the product titer.

Box 2 **Importance of precursors for shikimate pathway derived products.**

The crucial role of PEP, which is as well precursor as also cofactor of the shikimate pathway, has already been elaborated in the first study on production of ccMA in *E. coli* (Niu et al., 2002), where a difference in theoretical maximum yields of 43% was determined, depending on the availability of PEP. In another early study on *E. coli*, where the PEP synthase (*pps*) was overexpressed, it was found that not only PEP levels need to be increased but also E4P limits the formation of DAHP and E4P levels should, therefore, be increased too, in particular by overexpressing transketolase (*tktA*) (Patnaik and Liao, 1994). This was elaborated in a study combining *tktA* overexpression with knockout of *pykA, pykF* and deactivation of the PTS to increase PEP availability, thus reaching on almost 20-fold increase in carbon partitioning to the shikimate pathway (Gosset et al., 1996). A recent flux analysis on shikimate production in *S. cerevisiae* study even concluded that in yeast rather E4P is the rate limiting precursor (Suástegui et al., 2016). In a related study, the xylose utilizing yeast *Scheffersomyces stipitis* (*Pichia stipitis*) was employed, which was also further optimized for E4P formation by overexpressing *TKL1*, as well as channeling flux to and accumulating carbon in shikimate (by means of *ARO4^K229L^* and *ARO1^D920A^*). This led to a final titer of 3.11 g/L (Gao et al., 2017), which corresponds to one of the highest carbon-yields achieved in a yeast-based system. That this is a meaningful strain construction strategy is further underlined by another recent study on shikimate production in *Corynebacterium glutamicum* (Kogure et al., 2016). Here also the PTS was disabled to increase PEP availability through disruption of the *ptsH* gene, while ATP driven carbon-source uptake was strengthened by overexpressing *iolT1* (myo-inositol permease), *glk* (glucokinase), and *ppgk* (polyphosphate glucokinase) and flux to PEP was increased by overexpressing *gapA* (glyceraldehyde-3-phosphate dehydrogenase), while loss to glycerol was inhibited by deletion of *hdpA* (DHAP phosphatase). E4P availability was increased by enhancing flux through the PPP by overexpressing *tkt* (transketolase) and *tal* (transaldolase), co-utilization of xylose and arabinose complemented this. Additional overexpression of the *aroG, aroB, aroD*, and *aroE* (DAHP synthase, DHQ synthase, DHQ dehydratase, and shikimate dehydrogenase) genes along the shikimate pathway as well as knockout of downstream pathways (*qsuD, qsuB*, and *aroK*) lead to an impressive shikimate titer of 141 g/L. The corresponding yield reached 2/3rd of the theoretical maximum, which is close to industrial feasibility and the current record for a compound in the biosynthesis of aromatic. The corresponding productivity was nearly 3 g/(L × h), which is the threshold for industrial standards. Growth-arrested cells even reached a productivity beyond 5 g/(L × h) (Kogure et al., 2016). Lately, the same group has diversified the *C. glutamicum* based microbial cell factory for aromatics—in a different product context rather the *pyk* was knocked out instead of disabling the PTS, besides other extensive modifications throughout the pathway (Kitade et al., 2018).

### Perspectives and Recommendations for Future Strain Development

Systems Biology has already begun to revolutionize Biotechnology by replacing trial and error driven Metabolic Engineering with rational approaches (Dai and Nielsen, 2015). In this context especially Metabolic Modeling has tremendous potential for *a priori* development of strain construction strategies The radical *in silico* developed knockout strategy for minimum-efficacy production (Averesch and Krömer, 2014; Williams et al., 2015) is also applicable to other shikimate pathway derived compounds that involve the release of PYR, i.e., anthranillic acid and pABA (given it is feasible in the respective organism). In brief, deletion of the pyruvate kinase in theory rewires the central metabolism, rerouting flux through the shikimate pathway as the only remaining means for the metabolism to feed PYR into the TCA-cycle. That is, only if flux through the shikimate pathway is high enough to replace the junction between glycolysis and TCA-cycle. This may become possible by combining production of all products from the shikimate pathway, which result in PYR formation while also significantly increasing flux to and through the shikimate pathway by all available means. Thus, PYR formation from the shikimate pathway may become high enough to drive central metabolism. If successful, this might allow evolutionary adaptation of a PYK knockout strain to higher aromatics production (by selecting for the fittest organism also the highest aromatics producer will be obtained, due to coupling of growth to product formation) and could engender the next generation of an aromatics producing strain. Especially recent *C. glutamicum* based strains (Kogure et al., 2016; Kubota et al., 2016; Kitade et al., 2018) deliver yields high enough to potentially allow this; again only in case the knockout strategy is applicable to the organism, which is not a given and needs to be verified individually; for example it cannot be transferred to *E. coli* (Averesch and Krömer, 2014). In this context, the exploration of other organisms as suitable hosts for production of aromatics could be worthwhile. For example, *Bacillus subtilis* as one of the big four industrial microbial workhorses has so far been ignored for production of aromatics. Very exiting could be the implementation of production of aromatics with PYR as a by-product in an organism that does by nature not have a pyruvate kinase, like *Acinetobacter baylyi*, where it was shown that heterologous expression of a pyruvate kinase in this organism increases the growth rate (Kannisto et al., 2014).

If evolutionary adaptation allows to significantly improve aromatics production, sequencing, determination of SNPs and characterizations of, e.g., new feedback-resistant enzymes [similar to what has been done in the Stephanopoulos group (Santos et al., 2012)] of the adapted strain may lead to identification of unknown bottlenecks and help overcoming these. Further, this may enable reverse engineering, i.e., application of the novel targets to existing production strains. That way also strains or pathways, which do not allow the direct application of the PYK knockout (i.e., all pathways that proceed via the chorismate mutase), would benefit from the new platform strain.

As molecular biology tools for most model organisms used in Metabolic Engineering are advanced and streamlined, CRISPR has often limited advantage over traditional genetic engineering techniques, such as homologous recombination. However, when precise regulation is needed, such as conditional silencing, CRISPRi (Qi et al., 2013) is often superior to other techniques like RNAi (Crook et al., 2014), as CRISPRi directly inhibits transcription, opposing to RNAi, which inhibits translation and is thus less tight. Tools regulating expression on translational level also impose a higher burden on the metabolism of the organism and are not universally applicable [RNAi is limited to eukaryotes, in prokaryotes sRNAs can be utilized for posttranscriptional gene-repression (Na et al., 2013)]. Further, no equivalent to CRISPRa exists, which has the opposite function, and allows the activation and conditional upregulation of genetic targets (Dominguez et al., 2015). CRISPRi may be especially useful in case constitutive application of the developed strain construction strategy is not feasible [e.g., lethal knockout (Williams et al., 2015)]. In particular, in the context of the knockout strategy described above, CRISPRi may be used for (simultaneous) silencing of *PTS* and *PYK* while CRISPRa could be used to upregulate key-bottleneck enzymes. This would minimize technical effort, while constituting a chance to circumvent challenging genetic intervention strategies.

## Author Contributions

NA and JK jointly conceived the study. NA reviewed the literature and extracted the data, drafted the manuscript, and performed supporting calculations. JK edited the manuscript and guided the calculations. Both authors read and approved the final manuscript.

## Conflict of Interest Statement

The authors declare that the research was conducted in the absence of any commercial or financial relationships that could be construed as a potential conflict of interest.
