# Supplementary material for: Metabolic Engineering of the Shikimate Pathway for Production of Aromatics and Derived Compounds—Present and Future Strain Construction Strategies
Source: Front Bioeng Biotechnol. 2018 Mar 26;6:32. doi: 10.3389/fbioe.2018.00032 (PMC5879953; doi:10.3389/fbioe.2018.00032)
Supplement: Supplementary file 1 [file data_sheet_1.PDF]

Assumptions and calculations for determination of yields and rates

The productivity was calculated from the quotient of the final titer and the production time. In case the specific rates were not given in the respective publications, they were calculated from the quotient of the productivity and the formed biomass. If only the optical density was given, the biomass was estimated based on published correlations between optical density and biomass (see below for factors). In case no data on the biomass was given, it was estimated based on published organism specific biomass yields on the respective substrate and the amount of consumed carbon source. If the amount of consumed carbon source was also not given, it was estimated as well, based on the product yield and titer (cf. supplementary file 2 for calculations).

Correlation between optical density (OD) and biomass (g_{CDW}/L):

- *E. coli*: CDW [g/L] = 0.56 × OD_{660} (Link et al., 2008)
- *C. glutamicum*: CDW [g/L] = 0.25 × OD_{600} (Koch-Koerfges et al., 2013)
- *P. putida*: CDW [g/L] = 0.486 × OD_{600} (Yu et al., 2017)
- *S. cerevisiae*: CDW [g/L] = 0.2 × OD_{660} (Krömer et al., 2013)

Experimental biomass yields:

- *E. coli*: 0.45 – 0.51 $g_{CDW}/g_{glucose}$ (Link et al., 2008;Galvanauskas et al., 2013), 0.033 $g_{CDW}/g_{glycerol}$ (Murarka et al., 2008)
- *C. glutamicum*: 0.5 $g_{CDW}/g_{glucose}$ (Wittmann, 2010)
- *P. putida*: 0.49 – 0.59 C-$mol_{Biomass}$/C-$mol_{Glucose}$ (≙ 0.4045 – 0.487 $g_{CDW}/g_{glucose}$) (Hintermayer, 2016;Hintermayer and Weuster-Botz, 2017), 0.61 – 0.86 C-$mol_{Biomass}$/C-$mol_{Glycerol}$ (≙ 0.4925 – 0.6944 $g_{CDW}/g_{glycerol}$) (Hintermayer, 2016;Hintermayer and Weuster-Botz, 2017)
- *S. cerevisiae*: 0. 55 $g_{CDW}/g_{glucose}$ (Galvanauskas et al., 2013)).

For an extra layer of confidence, the biomass yields were also computationally determined by means of elementary flux mode analysis (Terzer and Stelling, 2008): For each organism and product, a metabolic network was compiled from literature (Melzer et al., 2009;Unrean, 2014;Beckers et al., 2016;Nils J. H. Averesch, 2017) (cf. supplementary file 3), amended with the respective metabolic pathway(s), as described in the respective studies and/or on KEGG. Elementary flux modes were calculated in MATLAB® (MathWorks®) using the most recent implementation FluxModeCalculator (van Klinken and Willems van Dijk, 2015) and evaluated as described before (Averesch and Krömer, 2014). Based on the achieved product yield in the respective study, the associated maximum biomass yields (maximum, as living organisms are likely to maximise their growth) were extracted from the distribution of flux modes. Biomass estimated based on computationally determined biomass yields was found to correlate well with the biomass estimated based on experimentally determined biomass yields in most cases (cf. supplementary file 2). Thus, in case no experimental data for the biomass yield was available in literature, calculations of the rates relied solely on the modelling data.

In this course, also the maximum theoretical product carbon yields for all the products were determined from the flux modes, by methods described before (Averesch and Krömer, 2014). All calculated data and calculations based on the data can be found in supplementary file 2.

Averesch, N.J.H., and Krömer, J.O. (2014). Tailoring strain construction strategies for muconic acid production in *S. cerevisiae* and *E. coli*. *Metab Eng Commun* 1**,** 19-28.

Beckers, V., Poblete-Castro, I., Tomasch, J., and Wittmann, C. (2016). Integrated analysis of gene expression and metabolic fluxes in PHA-producing *Pseudomonas putida* grown on glycerol. *Microb Cell Fac* 15**,** 73.

Galvanauskas, V., Grigs, O., Vanags, J., Dubencovs, K., and Stepanova, V. (2013). Model-based optimization and pO2 control of fed-batch *Escherichia coli* and *Saccharomyces cerevisiae* cultivation processes. *Eng Life Sci* 13**,** 172-184.

Hintermayer, S.B. (2016). *Charakterisierung der Anodenatmung von Pseudomonas putida in Rührkesselreaktoren.* Doktors der Naturwissenschaften Dissertation, Technischen Universität München.

Hintermayer, S.B., and Weuster-Botz, D. (2017). Experimental validation of in silico estimated biomass yields of *Pseudomonas putida* KT2440. *Biotechnol J* 12**,** 1600720-n/a.

Koch-Koerfges, A., Pfelzer, N., Platzen, L., Oldiges, M., and Bott, M. (2013). Conversion of *Corynebacterium glutamicum* from an aerobic respiring to an aerobic fermenting bacterium by inactivation of the respiratory chain. *Biochim Biophys Acta* 1827**,** 699-708.

Krömer, J.O., Nunez-Bernal, D., Averesch, N.J.H., Hampe, J., Varela, J., and Varela, C. (2013). Production of aromatics in *Saccharomyces cerevisiae*—A feasibility study. *J Biotechnol* 163**,** 184-193.

Link, H., Anselment, B., and Weuster-Botz, D. (2008). Leakage of adenylates during cold methanol/glycerol quenching of *Escherichia coli*. *Metabolomics* 4**,** 240-247.

Melzer, G., Esfandabadi, M.E., Franco-Lara, E., and Wittmann, C. (2009). Flux Design: *In silico* design of cell factories based on correlation of pathway fluxes to desired properties. *BMC Syst Biol* 3**,** 120.

Murarka, A., Dharmadi, Y., Yazdani, S.S., and Gonzalez, R. (2008). Fermentative Utilization of Glycerol by *Escherichia coli* and Its Implications for the Production of Fuels and Chemicals. *Appl Environ Microbiol* 74**,** 1124-1135.

Nils J. H. Averesch, V.S.M., Lars K. Nielsen, Jens O. Krömer (2017). Towards synthetic biology strategies for adipic acid production – an *in-silico* tool for combined thermodynamics and stoichiometric analysis of metabolic networks. *ACS Synth Biol*.

Terzer, M., and Stelling, J. (2008). Large-scale computation of elementary flux modes with bit pattern trees. *Bioinformatics* 24**,** 2229-2235.

Unrean, P. (2014). Pathway analysis of *Pichia pastoris* to elucidate methanol metabolism and its regulation for production of recombinant proteins. *Biotechnol Prog* 30**,** 28-37.

Van Klinken, J.B., and Willems Van Dijk, K. (2015). FluxModeCalculator: an efficient tool for large-scale flux mode computation. *Bioinformatics*.

Wittmann, C. (2010). "Analysis and Engineering of Metabolic Pathway Fluxes in *Corynebacterium glutamicum*," in *Biosystems Engineering I: Creating Superior Biocatalysts,* eds. C. Wittmann & R. Krull. (Berlin, Heidelberg: Springer Berlin Heidelberg), 21-49.

Yu, S., Lai, B., Plan, M.R., Hodson, M.P., Lestari, E.A., Song, H., and Krömer, J.O. (2017). Improved performance of *Pseudomonas putida* in a bioelectrochemical system through overexpression of periplasmic glucose dehydrogenase. *Biotechnol Bioeng***,** n/a-n/a.
